# Edge Computing Based IoT Architecture for Low Cost Air Pollution Monitoring Systems: A Comprehensive System Analysis, Design Considerations & Development

**DOI:** 10.3390/s18093021

**Published:** 2018-09-10

**Authors:** Zeba Idrees, Zhuo Zou, Lirong Zheng

**Affiliations:** School of Information Science and Engineering, Fudan University, Shanghai 200433, China; izeba17@fudan.edu.cn

**Keywords:** air pollution monitoring, IoT, edge computing, pollution sensors, electrochemical gas sensors

## Abstract

With the swift growth in commerce and transportation in the modern civilization, much attention has been paid to air quality monitoring, however existing monitoring systems are unable to provide sufficient spatial and temporal resolutions of the data with cost efficient and real time solutions. In this paper we have investigated the issues, infrastructure, computational complexity, and procedures of designing and implementing real-time air quality monitoring systems. To daze the defects of the existing monitoring systems and to decrease the overall cost, this paper devised a novel approach to implement the air quality monitoring system, employing the edge-computing based Internet-of-Things (IoT). In the proposed method, sensors gather the air quality data in real time and transmit it to the edge computing device that performs necessary processing and analysis. The complete infrastructure & prototype for evaluation is developed over the Arduino board and IBM Watson IoT platform. Our model is structured in such a way that it reduces the computational burden over sensing nodes (reduced to 70%) that is battery powered and balanced it with edge computing device that has its local data base and can be powered up directly as it is deployed indoor. Algorithms were employed to avoid temporary errors in low cost sensor, and to manage cross sensitivity problems. Automatic calibration is set up to ensure the accuracy of the sensors reporting, hence achieving data accuracy around 75–80% under different circumstances. In addition, a data transmission strategy is applied to minimize the redundant network traffic and power consumption. Our model acquires a power consumption reduction up to 23% with a significant low cost. Experimental evaluations were performed under different scenarios to validate the system’s effectiveness.

## 1. Introduction

Air quality is one of the key measures to be closely observed in real-time for today’s urban environments, because it has a paramount impact on human health, safety and comfort. Countries have established their own structures, policies and standards to monitor air pollution and generate alerts for inhabitants [1]. Although this information is restricted to outdoor environments, most of the measurements are static and only obtain average values; however in real time, air quality is variable and may be influenced by diverse circumstances [2], for example wind speed, population density, pollutant distribution, and whether the location is indoors or outdoors.

Conventionally, air pollution monitoring stations are large in sizes and expensive for installation and maintenance [3]. However, the air quality data generated by these stations is very accurate. Efforts have been made for alternative & cost efficient solutions. Internet-of Things (IoT) is a novel technology which attracts attention from both academia and industry. To overcome the flaws of current monitoring systems and their recognition methods and reduce the overall cost, this paper offers a novel approach that combines the IoT technology with environment monitoring [4,5,6]. This approach provides a low cost, accurate, easy to deploy, scalable and user friendly system. 

This paper presents a comprehensive review of pollution monitoring needs, existing monitoring systems, their limitations, and current challenges faced by these monitoring systems. We examine in depth the issues, infrastructure, data processing, and encounters of designing and deploying an integrated sensing node for observing indoor/outdoor air pollution. This project designed an air monitoring system model utilizing the edge computing & IoT architecture, assuring measurement accuracy and power efficiency with minimum cost.

To evaluate the system’s viability, a prototype was developed with the Arduino platform and experimental evaluation has been conducted in different sets. The rest of the paper is organized as follows: The background and previous research are discussed in Section 2. Section 3 describes the planned edge computing based IoT architecture. Section 4 explains the system implementation. Experimental evaluation and results are discussed in Section 5. Section 6 presents the conclusion.

## 2. Background and Related Work

With the rapid growth of the industrial sectors and urbanization, the environment becomes highly polluted to the level of disturbing the daily life of the people. Environmental pollution in the broad sense includes the pollution of air, water and the land. Of all these kinds of pollution, air pollution occupies the most prominent place in damaging the health of the people [7,8]. To reduce the impact of air pollution on individuals, the global environment and the global economy, great efforts have been made on air monitoring.

**Figure 1:**
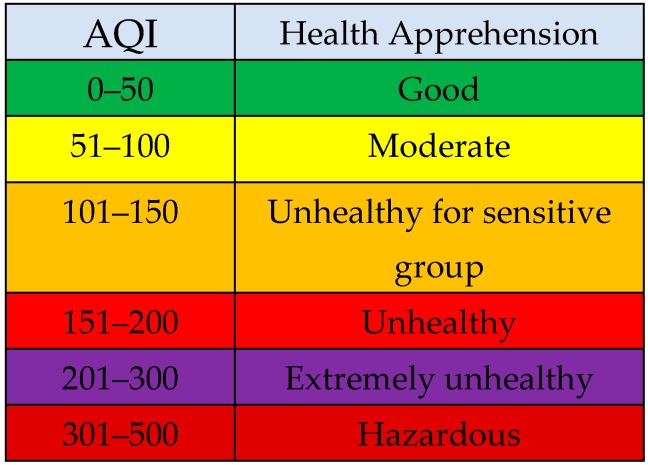
AQI levels according to China and the US Environmental Protection Agency.

- **Pollution Monitoring Systems and Related Requirements:** Air quality monitoring systems (AQMS) can be categorized as the indoor and outdoor pollution monitoring reliant on the place where the event occurs. Outdoor air pollution refers to the open and industrial environment. In contrast, the indoor case is the pollution of the air in small confined spaces within homes, work places, offices and closed areas like underground shopping centers and subways [9,10]. Because of their different environment and pollutant types, monitoring systems for indoor and outdoor air have different related requirements as described in Table 1.
- **Air Quality Index:** As a standard of measurement of air quality, AQI is a quantitative depiction of the air pollution level. The major pollutants involved in the analysis (as described by the Environmental protection agency US [11]) include fine particulate matter (PM_2.5_), inhalable particles (PM_10_), SO_2_, NO_2_, O_3_, CO. Here PM_2.5_ and PM_10_ are measured in micrograms per cubic meter (μg/m^3^), CO in parts per million (ppm), SO_2_, NO_2_, and O_3_ in parts per billion (ppb). AQI is divided into six levels in total, with green indicating the best and maroon the worst case [12,13], as shown in Figure 1 below.
- **Design and Deployment Strategies:** To obtain reliable and accurate data, conventional monitoring systems use complex measurement algorithms and various supplementary tools. As a result, these apparatuses are usually very high in cost and power consumption, and large in size & weight. Technical advancements resolve these issues to some extent, in that low cost ambient sensors with a small size and quick response are easily available. However, they cannot achieve similar data precision levels as conventional monitoring devices.

**Figure 2:**
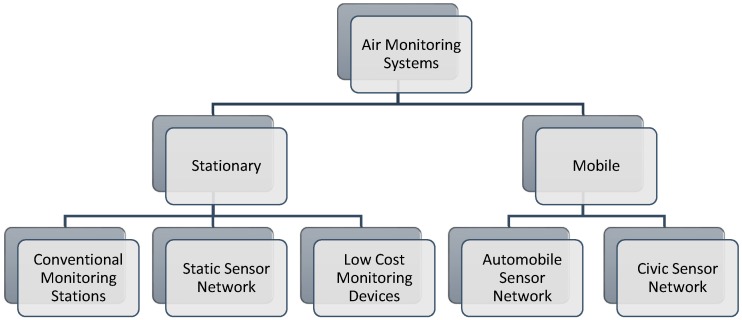
Air monitoring system’s categories presented in the recent literature.

Research shows that air monitoring systems are trending towards a new approach that combines low-cost sensors and the Wireless Sensor Network (WSN) into one system [3]. Low cost sensor based models help investigators recognize the dispersal of the air pollutants more competently and precisely. Even community users can evaluate their personal exposure to pollutants by means of wearable sensor nodes [14,15,16]. There have been numerous methodologies for air quality monitoring, as reported in recent research. They are mainly classified into two categories, i.e., stationary and mobile air monitoring. Figure 2 depicts the categories of the monitoring system based on deployment strategies in a hierarchical manner.

### 2.1. Related Work in the Literature

Reference [17] proposes an Internet of Things (IoT) system that monitors air pollution in real-time. The authors use multiple gas sensors, but do not follow the specific standard measurement procedure. Also there is no proper architecture for system and it lacks experimental evaluations. Reference [18] designed a monitoring system based on the IoT concept where they monitor the environmental parameters using low cost sensors from MQ series, there is no data validation or the sensors calibration procedure that is compulsory in case of low cost sensors.

The system presented in Reference [19] operates on an existing Wi-Fi network via the MQTT protocol. Their intention was to monitor the indoor air quality, and the focus was on a single pollutant, being PM control. Reference [8] aims to detect PM only and notify the user via email using a low cost dust sensor and Raspberry Pi for data transmission. Reference [20] put their efforts into developing a system to measure the 3D AQI map. The researchers prototype a quad copter for 3D data collection and compare the results with official data and claim to achieve reasonable accuracy. This design is not cost efficient and the system implementation is quite complex.

Reference [21] uses LTE network communication and IoT concept for air pollution monitoring, they does not consider the limitations of the low cost sensors and compare their results with official data provided by National Ambient Air Quality Monitoring Information System. Reference [22] presented a mobile air quality monitoring system to attain a low-cost solution. They used a public bus rooftop as a sensor carrier. In case of mobile sensing, many considerations need to be addressed for the sensors to work properly, as the sensors work well in the static form [3]. While the cost of the system is low, due to lack of system design parameters, the data is not very reliable. References [23,24] conducted a comprehensive study for sensor network establishment in pollution monitoring systems. They analyzed sensor node placement strategies and issues. This work was evaluated via simulations.

Most of the existing systems discussed above place their emphasis only on one or two main pollutants; if the systems measured more pollutants, then the data processing capabilities were unclear. While they used low cost sensors, they lack a calibration mechanism and do not deal with sensor drift compensation issues other than in the work presented in Reference [25]. Furthermore, there were no power management strategies for these systems. Table 2 presents the summary of recent efforts in the field of air pollution monitoring by comparing their design strategies, protocols, hardware/software tools and costs. 

### 2.2. AQMS Challenges

In recent years, many advanced air quality monitoring systems have been proposed, as discussed in Section 2.1. There are still various subjects that are worth discussing, a few of them are considered below.

- **Cost & Maintains:** The cost of the instruments used in conventional monitoring systems is approx. 90,000 USD [3]. A typical air quality measurement station requires about 200,000 USD for construction and 30,000 USD per year for maintenance [9]. The inclusive cost of the sensor network is also highly reliant on the sensors category and number of deployed nodes. As such, there is a vital need to make the system cost-efficient.
- **Accuracy:** While the expensive monitoring stations are hard to maintain, the data quality and precision is very high. The systems with low cost tools have poor accuracy, so obtaining precision in these systems is a major challenge.
- **3D Data Attainment:** The majority of the systems published in literature are merely capable of monitoring the air quality of the urban surface or roadside, whereas the inevitabilities and significance of the 3-Dimensional air pollution statistics are portrayed in Reference [3]. Satellites-based 3-Dimensional monitoring systems deal with similar issues to conventional monitoring systems. Reference [20] applied efforts to acquire the 3-Dimensional data in real time. However, the system cost and power consumption is very high.
- **Absence of Active Monitoring:** The sensing modules in SSN, VSN and ASN systems update the data periodically and are all passive monitoring systems. Active monitoring could offer greater flexibility and quality for the service.
- **Flexibility/Scalability:** Literature studies realize that the majority of the existing systems have no ability to add-on hardware & software reconfigurations that are required when the sensing node classes are revised. In practical scenarios with large-scale applications, there are huge numbers of sensor nodes in the system with an add-on ability that are essential in this case.
- **Power Consumption:** Power consumption is not a major issue for indoor air monitoring systems other than in the case of cost concerns. But for the outdoor systems and especially for the sensor network-based approaches, power consumption is an important design consideration [25]. The energy sources could be restocked via solar or other methods. As the size of the network grows, this task becomes more difficult and remains an open research challenge.

Hence all the discussed challenges and the issues can be resolved to some extent by using advanced technologies. These issues should be considered carefully while designing air monitoring systems. 

## 3. Proposed Edge Computing Based IoT Architecture for the Air Pollution Monitoring

**Figure 3:**
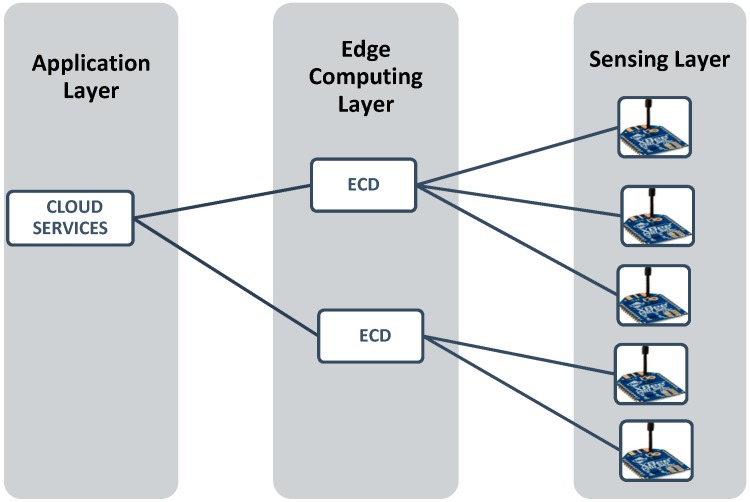
System architecture.

We have designed an edge-computing based IoT architecture for the air pollution monitoring system. As demonstrated in Figure 3, a layered IoT architecture is employed in the monitoring system. Three layers were defined as the sensing layer, network layer and the application layer, respectively [39]. All the layers are communicating via Zigbee and Wi-Fi, although any other similar technology can be used for this purpose. The total work load is balanced and distributed over these three layers according to the edge-computing mechanism [40,41].

- **Sensing Layer:** This is the basis of the whole monitoring system. The main responsibility of this layer is to sense the air quality. The sensing nodes are the main entities of this layer and can be deployed over the wide area. Hardware and software details about these nodes are provided in the next section.
- **Edge Computing Layer:** This layer is composed of edge-computing devices (IoT gateways). Its duty is to communicate with the other two layers. ECD gathers the data from the entire sensing layer and after necessary processing, passes the data to the application layer.
- **Application Layer:** The application layer is responsible for providing collaborative services to the consumers and the data storage. It can be distributed into two chunks: The IoT cloud (IBM cloud), and user applications. Once it receives the data reported from the edge computing device, it stores the data in the database of the cloud and provides data visualization in numerous ways, as detailed in Section 3.2.

### 3.1. System Implementation 

System implementation comprises of the hardware and software parts, with the detailed description given below.

#### 3.1.1. Hardware Prototype

**Figure 4:**
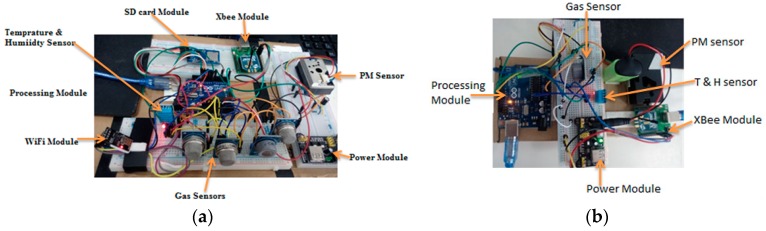
(**a**) ECD module. (**b**) Sensing module.

The hardware of the system primarily includes the sensing module (SM) and an edge computing device (ECD), as shown in Figure 4a,b respectively. The sensing node collects the real-time air pollution information and passes this data to the ECD through a wireless channel. A detailed description of these two modules is presented as follows.

**Figure 5:**
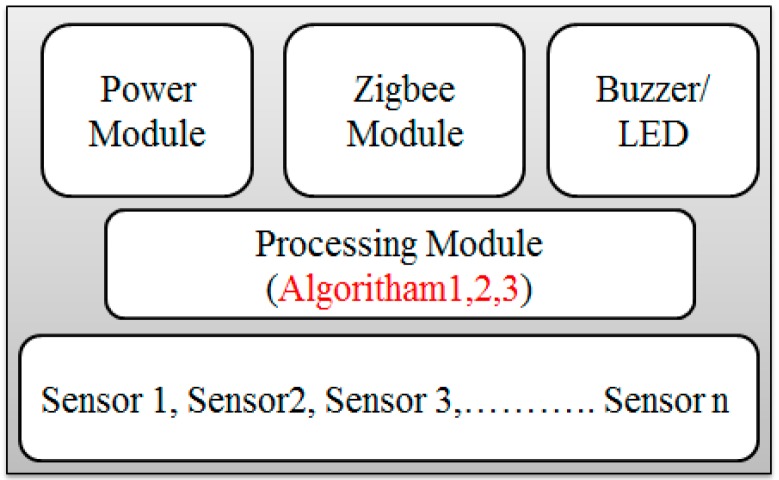
Sensing Module.

**Figure 6:**
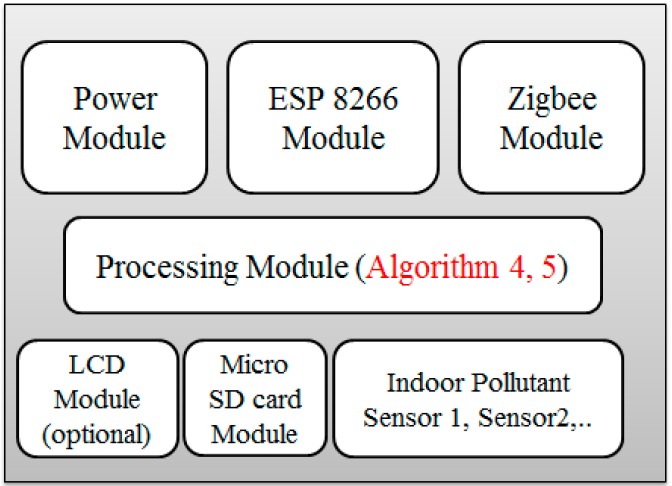
Edge computing device.

- **Sensing Module:** As shown in Figure 5, each SM is equipped with four sub modules, which are the sensor block, processing module, communication module and the power module.
  A *Sensor Block*: To make the system cost efficient, we use the low-cost sensor for smaller pollutants and more accurate sensors for the major pollutants present in the air. Deprived of the complex process, these sensors can measure the air quality in a few seconds. Although the precision of these sensors may not be comparable to conventional monitoring stations, it is sufficiently effective to demonstrate the trend of the air quality level. This module is designed to possess six different sensors, GP2Y1014AU0F, DSM501, MQ-7, GSNT11, SO2-AF, and MiCS2610-11 for the detection of PM_10_, PM_2.5_, CO, NO_2_, SO_2_ and O_3_. Additionally, a DHT11 humidity & temperature sensor was installed to resolve temperature and the humidity dependency.
  B *Processing Module*: Sensors pass their raw data to processing modules such as ATmega328P. This module performs the necessary processing on the data that is presented in Section 4 as the calibration the power management algorithms.
  C *Communication Module*: The Zigbee/802.15.4 protocol was used to communicate/exchange the data between the sensing module and ECD. XBee S2C 802.15.4 RF Modules are used for this purpose, this module provides quick, robust communication in point-to-point, peer-to-peer, and multipoint/star configurations. The module has the following features: 2.4 GHz for worldwide deployment, sleep current of sub 1 μA, with a Data Rate RF of 250 Kbps, a serial of up to 1 Mbps, an indoor/urban range of 200-ft. (60 m) 300-ft. (90 m), and an outdoor/RF line-of-sight range of 4000-ft. (1200 m) 2 miles (3200 m).
  D *Power Module*: The power module consisting of a rechargeable lithium battery and the control module. The battery is connected to the control module that converts 9 v to 5 v and 3 v. This can provide a safe and stable power supply for other sub modules.
- **Edge Computing Device:** As shown in Figure 3, the ECD in on the second layer that is the network layer and is obtained using the Arduino platform. For the purpose of simplicity and to keep the costs low, this platform is meeting the current prototype requirements, although any other advance and more powerful board can be used for device development. Block diagram of the ECD is shown in Figure 6 and sub modules are described below.
  A *Processing module*: This module is responsible for calculating the AQI, data analysis and power management. To prototype, the local data-based SD card was integrated with a device that stores hourly data, with only the daily AQI or AQI sliding window being posted to the cloud, as it saves power and communication bandwidth. This system is scalable and can be configured to multiple modes, including daily, hourly, and sliding windows modes. A sliding window case is where the AQI will be posted to the cloud only when it varies enough to change the range window which is depicted in Figure 1. Mode selection is dependent upon the application and user demand.
  B *Sensor Module*: This module contains low cost electrochemical sensors from the MQ series (MQ-135, MQ-6, MQ-7, MQ-9) for measuring indoor pollutants and hazardous gases [10,35]. The GP2Y1014AU0F, was installed to measure the dust & particle matters. Additionally, a DHT11 humidity and temperature sensor was installed to resolve temperature and humidity dependency.
  C *Communication Modules*: ECD device include two communication modules, one is an XBee S2C module to communicate with SM and the other is a Wi-Fi ESP8266 used to interface with the cloud platform.

### 3.2. Software Implementation

**Figure 7:**
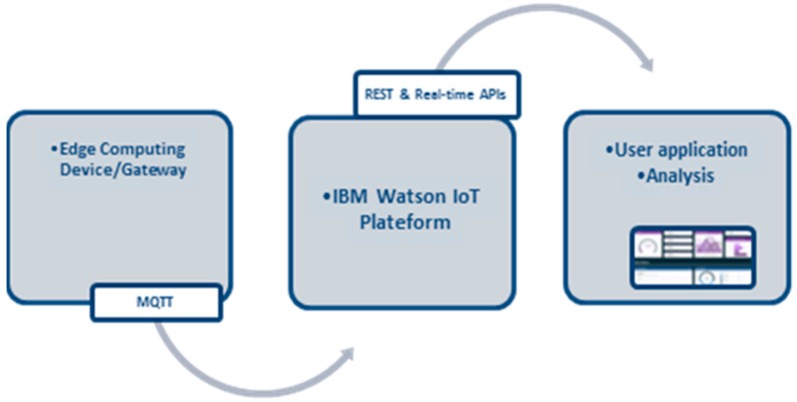
System software architecture.

The large number of software programs are required for both the IoT cloud and the client so that consumers can enjoy full services. There are three primary kinds of servers in the IoT cloud with diverse functionalities, i.e., Data Processing Server, Storage Server, and HTTP Server. We progress the design considerations and plan a real-time ample air-quality level indication scheme, which efficiently manages vibrant changes. IBM Bluemix was used to implement these services. The overall software architecture is described in Figure 7.

**Figure 8:**
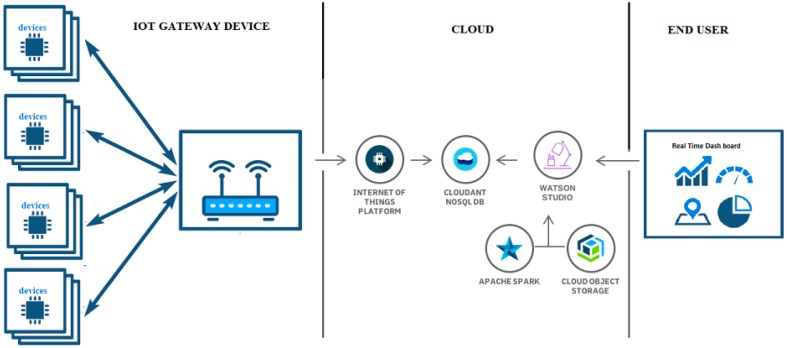
IoT architecture employing cloud services.

**Figure 9:**
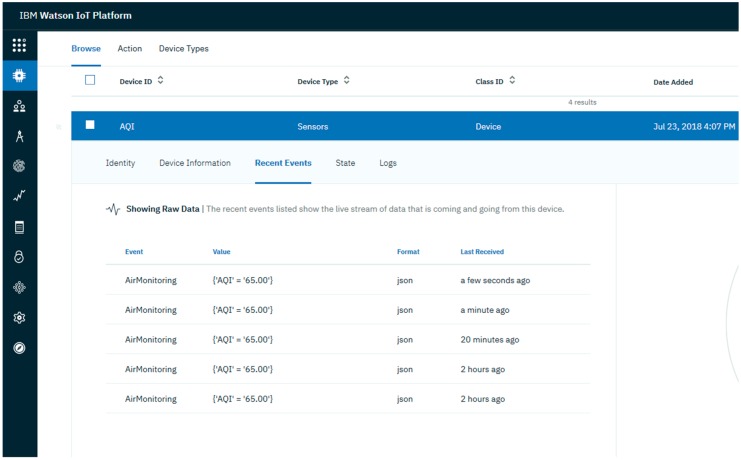
IBM Watson IoT platform for data visualization.

- **IoT Platform and Cloudant Data Base:** The IoT platform communicates with ECD and collects the data from it. It uses the built-in web console dashboards to screen data and analyze it in real time. Users can define rules for monitoring circumstances and triggering actions that include alerts, email notifications, Node-RED flows, and other services reacting quickly to dangerous changes. Figure 8 provides a diagram of the architecture.
- **Cloudant Data Base:** Offers access to a NoSQL JSON data layer. This is compatible with CouchDB and is manageable through the HTTP interface for web application models. Every document in the database is accessible as JSON via a URL, and data can be retrieved, stored, or deleted individually or in bulk. Data sets were analyzed to disclose the air pollution trends. Analytical results were stored in the NoSQL data base.
- **User Application:** Either a website or a mobile application can be used to exhibit the air quality facts to the consumer. Watson studio was used to explore air quality data and create visualizations for the end user. Watson Studio offers a collection of tools and a cooperative environment for data scientists, developers and domain specialists. Using the presentation applications, the AQI could be displayed in real-time, e.g., current AQI indication and trends for the present day/week/month. In addition, the trends for the individual pollutants can also be displayed in a graphical view. Other available visualizations include current status, recent events, and data logs, an example is shown in Figure 9.

## 4. Data Processing

### 4.1. Pre-Calibration

Characteristics of the low-cost sensor differ from sensor to sensor and from production lot to production lot. Thus, every sensor needs pre-calibration to accurately measure gas capacity. Algorithm 1 shows the steps for pre-calibration, here coefficient a and b are extrapolated from the curves provided in the sensors data sheet, for example characteristic curve for the MQ 135 sensor can be taken from Reference [42].


**Algorithm 1: Pre-Calibration for Gas Sensors**
1: Calculation of *R*_0_ (sensor resistance in the clean air) 2: Calculation of *R*_s_ (sensor resistance presence of certain gas) 3: Analog read sensor pin 4: Take multiple samples and calculate the average (S) 5: *R*_0_ = S/clean air factor 6: Extrapolate coefficients a and b 7: Calculate ppm, ppm =a×(RsR0)b


### 4.2. Auto Calibration (Temperature & Humidity Dependency)

Low cost sensors are often affected by temperature and humidity, as described in their data sheets [42,43,44,45]. Therefore, pre-calibrated values require to be adjusted with respect to the temperature and humidity dependency to ensure sensing accuracy [25]. Algorithm 2 details how to apply the auto-calibration. The calibrated value of the gas sensors $C_{v}$, is calculated as:

(1)  Cv=Rs R0× DTH

where $R_{s}$ is the sensor resistance in the presence of a certain gas, $R_{0}\;$ is the sensor resistance in clean air, and *D_TH_* is the temperature and humidity dependence value for the calibration. The ratio of the sensor resistances, *Rs*/*R*_0_, is obtained by:

(2)  Rs R0=(Rl×AmAv)−Rl

where $R_{l}$ is the external resistance, temperature and the humidity dependence value for the calibration, $D_{TH}$, can be obtained by:

(3) $$\;D_{TH\;}=\gamma t^{2}-t+\delta$$

where *t* is the current temperature and δ  is the humidity dependency value.


**Algorithm 2: Auto Calibration for Temperature and the Humidity Dependency**
$C_{v}\;$(Calibrated data of the gas sensors), *t* (Temperature), $H_{r}$ (Humidity Value), $A_{v}$ (Current Analog read value of gas sensor), $R_{l}\;$ (The external load resistance), $A_{m}$ (The maximum analog read value of a), RsR0 (resistance ratio) 1: Read sensors data **(**$A_{v}$, *t*, and $H_{r}$**)** 2: Convert the measured values to dependency values ($H_{r}$, *γ*, and *ψ*) 3: Calculate the value of temperature and humidity dependency, $D_{TH}$ with Equation (3) 4: Calculate the RsR0 using (2) 5: Calculate the calibrated value $C_{v}$ using (1)

### 4.3. Data Smoothing Algorithm

According to Reference [46], the measurements that significantly deviate from the normal pattern of the measured data are called outliers. They need to be detected and removed to obtain the accurate data. A data smoothing algorithm is employed to filtering out this noise. To smooth the gas sensor data, important data trends were recorded in a repeated statistical manner and standard deviation with ±3*σ* is used for limitation. Algorithm 3 explains the smoothing algorithm.


**Algorithm 3: Sensor Data Smoothing**
1: *R_s_ (Sensor Resistor)* 2: *R_l_ (Load Resistor)* 3: *R_s_/R_l_* 4: **if**
*R_s_/R_l_* ≥ ±4*£(R_s_/R_l_)* 5: **then** Perform filtration 6: **else** Keep *R_s_/R_l_* 7: **end if**

### 4.4. Data Transmission Strategy

An Algorithm 4 was implemented to reduce the power consumption at the sensing node and the ECD. The key idea behind this algorithm is that the data will only be transferred if it is useful. The sensing node only transmits the data to ECD if the measured value was significantly different to the previous value and the amount of difference is specified by the ∆. The ECD calculates the AQI and updates it to the cloud only if the AQI changes its window. If the variation of the AQI is within the same window, it will not be posted to the cloud (on an hourly update case), For example if the AQI varies from 51 to 100, it remains in the moderate window and if the value crosses the limit and moves to the next window, it will be updated. Algorithm 5 describes the multiple SM scenarios and the inclusion of their individual AQI to enhance the data accuracy.


**Algorithm 4: Data Transmition Strategy for ECD and SN**
*For ECD* **$\;I_{AQ.t}=\max\left\{I_{1},\;I_{2},\;I_{3}\ldots \ldots \ldots .I_{n}\right\}$** $IAQ.tIAQ.tI_{AQ.t}$ (Overall Air quality index at time *t*) 1: if $I_{AQ.t}=I_{AQ.t-1}$ 2: then *Don’t send to cloud/user app* 3: else 4: if $I_{AQ.t}\neq I_{AQ.t-1}$ 5: if $I_{AQ.t}>\alpha I_{AQ.t-1}\;\|I_{AQ.t}<\beta I_{AQ.t-1}$ 6: then Update the AQI on the cloud/user end *For SN* **$\;I_{n}=\frac{I_{h}-I_{l}}{CB_{h}-CB_{l}}\left(C_{t}-CB_{l}\right)+I_{l}$** $I_{n.t}$ (Overall Air quality index at time *t)* 1: if $I_{n.t}=I_{n.t-1}$ 2: then *Don’t send data to ECD* 3: else 4: if $\;I_{n.t}\neq I_{n.t-1}$ 5: if $\;I_{n.t}>\alpha I_{n.t-1}\;\|I_{n.t}<\beta I_{n.t-1}$ 6: then Update the AQI on the cloud/user end 


**Algorithm 5: Enhancing the Efficiency of the Overall AQI**
**$\;I_{AQI.1}=\max\left\{I_{1},\;I_{2},\;I_{3}\ldots \ldots \ldots .I_{n}\right\}$** $I_{AQI.N}\;$(Overall Air quality index from node *N* at time *t*) 1: Average calculation of individual AQI of each pollutant $I_{n}=\frac{I_{a}+I_{b}\ldots \ldots ..I_{N}}{N}$ 2: Calculation of overall AQI $I_{AQI.1}$ 3: Average calculation of AQI from each Sensing Node $I_{AQI.N}$ $I_{AQI.2}=\frac{I_{a}+I_{b}\ldots \ldots ..I_{N}}{N}$ 4: if $I_{AQI.2}$**$\text{>}I_{AQI.1}$** 5: then: **$I_{AQI}=:\;I_{AQI.2}$** 6: else 7: if **$I_{AQI}=:\;I_{AQI.1}$**

### 4.5. Power Consumption Analysis & Computational Cost

The energy consumption used by the whole system is the addition of the energy consumed by each task. The average power is computed by considering the consumed power and the application specific period. $P_{basic}$ is the power consumption in the “idle” state. The execution time of the specific task $\tau_{task}$ is estimated by direct measurements or by timing estimation tools. Below is the power consumption analysis for a single sensing node over one day with and without applying a system model. Here $P_{Sensors}$ is the power consumed by the pollution sensors and has a fixed value. $P_{Procesing}$ represents the total processing power of the Arduino. $P_{Communication}$ consists of the power required to communicate with ECD. In our case, it only counts the number of transmissions from sensing the module to ECD. XBee module requires 1 mw of transmission power denoted as ѱ. Arduino consumes an average power of 734 mw denoted as $P_{basic}$. In Equation (6) $\tau_{task}$ is the execution time of the particular task.

(4) $$\;TPC_{S.N\;}\;=P_{Sensors}\;+P_{Procesing}\;+P_{Communication}\;$$

(5) $$\;P_{Procesing\;}\;<\;P_{Communication}<P_{Sensors}\;$$

(6) $$\;P_{Procesing\;}=P_{basic}\times \tau_{task}$$

(7) $$\;P_{Sensors\;}=\overset{n}{\underset{i=1}{\sum }}S_{i}$$

(8) $$\;P_{Communication\;}=\overset{n}{\underset{i=1}{\sum }}T_{i}+R_{i}$$

The measurement interval depends upon the type of sensor, the response time and the sensing algorithm. The response time is different for the different sensors, as summarized in Reference [3]. We took the measurements every second for particular matter, and every minute for the gases. In the daily case, the individual AQI for CO & O_3_ in ppm (parts per million) is calculated over 8 h, for SO_2_ & NO_2_ in ppb (parts per billion) over 1 h and finally the PM concentration in μg/m^3^ is calculated over 24 h. Therefore the number of transmissions were calculated accordingly, with 86,400 (transmission every second, for 24 h), 480 (transmission every minute for 8 h), and 60 (transmission every minute for 1 h). 

**Case1** (without employing the designed methodologies): Here ${£}_{PM2.5}\;$ is the number of transmissions required for PM_2.5_, ${£}_{PM10}$ for PM_10_ and $\alpha_{CO}\;,\alpha_{NO2}\;,\;\alpha_{SO2}\;,\;\alpha_{O3}$ for CO, NO_2_, SO_2_, O_3_ respectively.

**A:** Hourly.

(9) $$\;TNT_{S.N\;}\;={£}_{PM2.5}{}^{}\;+{£}_{PM10}{}^{}\;+\alpha_{CO}{}^{}+\alpha_{NO2}{}^{}+\alpha_{SO2}{}^{}+\alpha_{O3}{}^{}$$

$$\;TNT_{S.N\;}{}^{}\;=(60\times 60_{})+{\left(60\times 60\right)}^{}+\left(1\times 60\right)+\left(1\times 60\right)+\left(1\times 60\right)+\left(1\times 60\right)$$

$$\;TNT_{S.N\;}{}^{}\;=3600_{}+3600^{}+60+60+60+60$$

$$\;TNT_{S.N\;}{}^{}\;=7440\;\left(per\;hour\right)$$

$$\;Total\;Power_{S.N\;}{}^{}\;=24\times 7440\left(per\;day\right)$$

$$\;Total\;Power_{S.N\;}{}^{}\;=178,560ѱ$$

**B:** Daily.

(10) $$\;TNT_{S.N\;}{}^{}\;={£}_{PM2.5}{}^{}\;+{£}_{PM10}{}^{}\;+\alpha_{CO}{}^{}+\alpha_{NO2}{}^{}+\alpha_{SO2}{}^{}+\alpha_{O3}{}^{}$$

$$\;TNT_{S.N\;}{}^{}\;={\left(24\times 60\times 60\right)}_{}+{\left(24\times 60\times 60\right)}^{}+\left(8\times 60\right)+\left(8\times 60\right)+\left(1\times 60\right)+\left(1\times 60\right)$$

$$\;TNT_{S.N\;}{}^{}\;=86,400_{}+86,400^{}+480+480+60+60$$

$$\;TNT_{S.N\;}{}^{}\;=173,880$$

$$\;Total\;Power_{S.N\;}{}^{}\;=173,880ѱ$$

**Case2** (with designed methodologies): here $C_{PM2.5}$ is the number of transmissions required for PM_2.5_ concentration, $C_{PM10}{}^{}$ for PM_10_ and $\rho_{CO}{}^{},\;\rho_{NO2}{}^{},\;\zeta_{SO2,}{}^{}\;\zeta_{O3}{}^{}$ for CO, NO_2_, SO_2_, O_3_ respectively.

**A:** Hourly.

(11) $$\;TNT_{S.N\;}{}^{}\;=C_{PM2.5}{}^{}\;+C_{PM10}{}^{}\;+\rho_{CO}{}^{}+\rho_{NO2}{}^{}+\zeta_{SO2}{}^{}+\zeta_{O3}{}^{}$$

$$\;TNT_{S.N\;}{}^{}\;={\left(1\times 24\right)}_{}+{\left(1\times 24\right)}^{}+\left(1\times 24\right)+\left(1\times 24\right)+\left(1\times 24\right)+\left(1\times 24\right)$$

$$\;TNT_{S.N\;}{}^{}\;=144$$

$$\;Total\;Power_{S.N\;}{}^{}\;=144ѱ\;\left(per\;day\right)$$

**B:** Daily.

(12) $$\;TNT_{S.N\;}{}^{}\;=C_{PM2.5}{}^{}\;+C_{PM10}{}^{}\;+\rho_{CO}{}^{}+\rho_{NO2}{}^{}+\zeta_{SO2}{}^{}+\zeta_{O3}{}^{}$$

$$\;TNT_{S.N\;}{}^{}\;=1_{}+1^{}+1+1+1+1$$

$$\;TNT_{S.N\;}{}^{}\;=6$$

$$\;Total\;Consumed\;Power_{S.N\;}{}^{}\;=6ѱ$$

 Total power saved (A)=178,416 ѱ

 Total power saved (B)=173,878 ѱ

The communication consumes around 35% of the total power, with a designed strategy this consumption reduces to 10% (25% reduction) at a cost of a small increase in processing power consumption (2% increase), hence saving 23% of the total power per node.

The total computational burden of the system primarily consists of five algorithms presented in Section 4. To balance the load between the sensing node and the ECD algorithm, 1–3 run on sensing node while 4 & 5 incorporated by the ECD that reduce the computational burden of the sensing node up to 70% approx. and saves significant power as mathematically described above.

## 5. Experimental Evaluation

In order to evaluate the effectiveness of the system, the sensing module, edge-computing device and IBM platform were integrated. As a metric to indicate the air quality, AQI $“I_{AQI}”$ is calculated by measuring six main pollutants as mentioned in Section 3.1.1. The critical points for the six air pollutants are given by China’s Ministry of Environmental Protection and other bodies in References [11,47,48]. A discrete score $I_{n}$ is allotted to the level of each pollutant as calculated by Equation (13) and the absolute AQI ($I_{AQI}$) is the highest of them as described by the Equation (14). 

(13) $$\;I_{n}=\frac{I_{h}-I_{l}\;}{CB_{h}-CB_{l}}\left(C_{t}-CB_{l}\right)+I_{l}$$

(14) $$\;I_{AQI\;}=\max\left\{I_{1},\;I_{2},\;I_{3}\ldots \ldots \ldots .I_{n}\right\}$$

$I_{n}$: Air-Quality Index of the $Nth^{}$ pollutant
$C_{t}$: Truncated concentration of the $Nth^{}$ pollutant
$CB_{l}$: The concentration breakpoint that is *≤*$C_{t}$
$CB_{h}$: The concentration breakpoint that is *≥*$C_{t}$
$l_{l}$: AQI w.r.t $CB_{l}$
$l_{h}$: AQI w.r.t $CB_{h}$

According to the above equations $I_{AQI}$ will be the individual AQI i.e., $I_{n}$ of that particular pollutant which acquires the highest value. According to the historical statistics of official AQI data, the major and dominant pollutant is PM_2.5_ [13,49]. $I_{PM}$ (Discrete AQI of PM_2.5_ and $I_{Gs}$ (discrete AQIs of other pollutants) are related as $I_{PM}\gg \;I_{GS}$. Keeping these statistics in mind, this prototype selected the three dominant pollutants PM_2.5,_ PM_10_ & CO to calculate the outdoor AQI. However the SM is scalable and can measure all six pollutants according to the requirement of the sensitive scenario. This selection reduces the cost of the sensing node without significantly affecting the data accuracy, as including more pollutants enhances the cost of the individual node and overall system [24].

The overall functionality of the system was demonstrated by conducting the experiments in different settings—a living room (small), office (medium), and an open environment. In the small-size living room (5 m × 3.5 m) it was estimated that one sensing node was adequate, which was placed in the middle side of the room at a height of 1.7 m. For the open environment, the effective height of the node was kept at 9 m. 

### Results and Discussions

**Figure 10:**
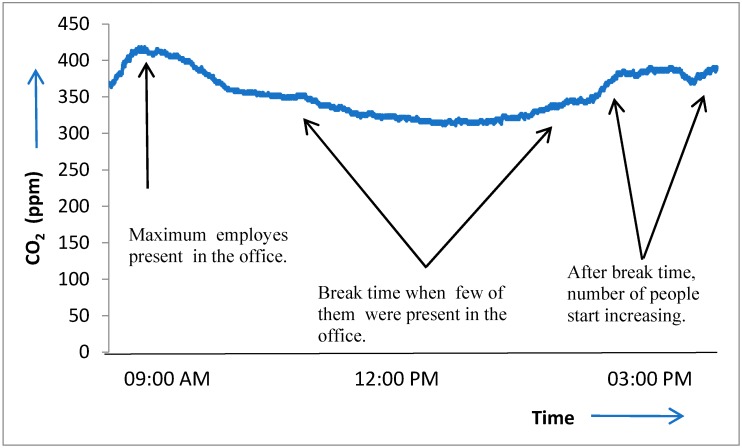
CO_2_ measurements over six hours in the office setting.

**Figure 11:**
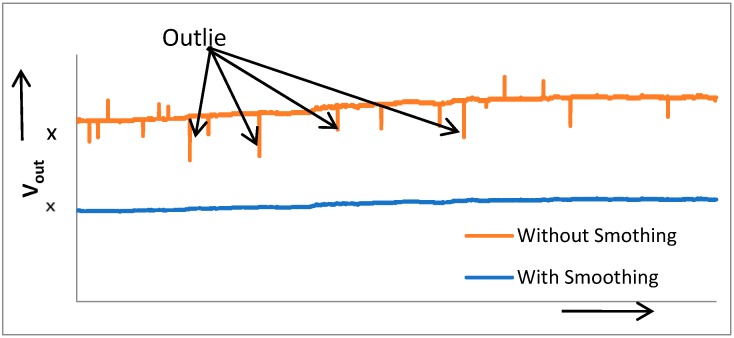
Sensors raw sata with and without smoothing algorithm.

To examine the viability of the system and the employed algorithms, measurements were taken with and without flattening the calibration and accumulation algorithms. MQ-135 could measure various gases as described in [42], for testing purpose it was calibrate to measure CO_2_ only. CO_2_ was chosen to demonstrate the effectiveness of the calibration algorithm due to the ease of the experimental setup and its plentiful availability in the target area. The data was collected over six hours in an office setting (13–17 employees), and Figure 10 depicts the results. Initially there were only four persons, with the passage of the time, the number of persons in the office start to increase, and the ascending level of CO_2_ indicate the sensor’s ability to detect that change. The CO_2_ ppm level reached its maximum value when around 15 persons were present in the office, as shown in the graph below. During the break time, CO_2_ ppm remained in its lower range, and data trends with time and scenario verified the calibration’s effectiveness. Outliers frequently occurred in real-time measurements and they needed to be detected and eliminated. Another test was performed to validate the smoothing algorithm in a small room (single cubical) setting over one hour. Minor variations were observed in V_out_ as numbers of persons were quite small as compare to office setting (1 vs. 15). Figure 11 shows the data measurements, graph contain raw data from the sensor with outliers. After applying the smoothing algorithm we get the clean data and outliers have been effectively removed. 

In the graph, x represents the value of V_out_ remaining the same before and after the smoothing algorithm. Data is plotted in the same graph purely for comparison, as the smoothing algorithm does not affect the readings except the abnormal ones that are indicated by the arrowheads.

For the air pollution monitoring evaluation, measurements have been conducted in the dormitory building of the FUDAN University from 21 April to 5 May 2018. We selected 15 consecutive days and 24 readings each day (every hour) to determine the consistency of the measurement. Every monitoring instance considered different environmental and weather parameters, such as temperature, relative humidity, and wind speed. 

**Figure 12:**
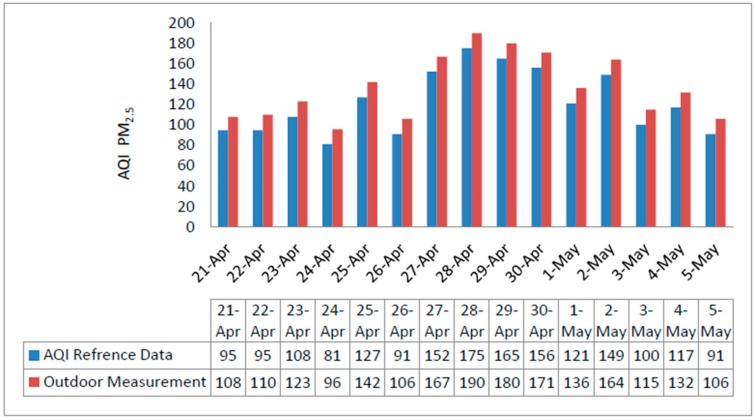
Outdoor measured PM_2.5_ AQI compared with official data, from 21 April to 5 May 2018.

**Figure 13:**
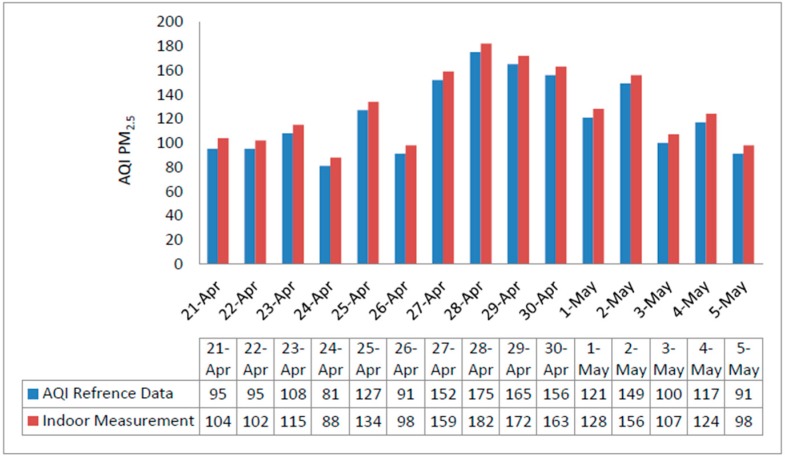
Indoor measured PM_2.5_ AQI value comparison with official data, for 15 days from 21 April to 5 May 2018.

Air monitoring data counting AQI for PM_2.5_ is shown in Figure 12. To validate the reliability of the system, additional data sets were acquired from recognized PM_2.5_ databases http://www.young-0.com/airquality/ [49]. These datasets are compared in Figure 12 and Figure 13 for outdoor and indoor environments respectively. 

**Figure 14:**
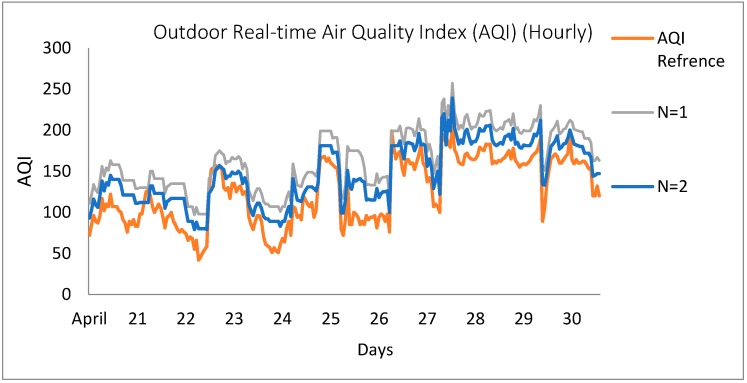
Comparison between the measurements with single and multiple sensing nodes (N = 1 & N = 2) from 21–30 April 2018.

It is apparent from Figure 14 that the trends of the AQI lines accord well when measured with multiple sensing nodes as compared to the single node case. Although the data trends were comparable with official data in the single node (N = 1) scenario, the gap and disparity is higher than in the multiple node (N = 2) case. It is clear that the sensing efficiency improves with the involvement of more sensing nodes because it enhances the quality of the data by realizing the sensor drift and enhancing the coverage capability. Additionally, the multiple node approach somehow combats the issue of the non-uniform pollution density. The discrepancy between the measurements and reference data may be due to several factors, such as the different measurement locations, respective environment, data acquisition techniques and the type of the incorporated sensors.

**Figure 15:**
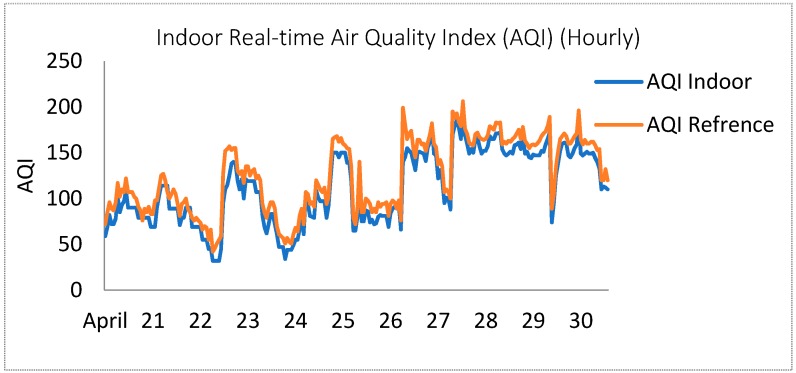
Comparison between the measured and reference data on overall AQI (Indoor) from 21–30 April 2018.

Figure 15 shows the AQI trends measured in the indoor environment. It can be seen that these measurements have less discrepancy as compared to the outdoor areas, this is due to the fact that environmental factors have less effects in the closed setting. Consequently, the air quality information acquired by this monitoring system was able to determine the accuracy and reliability necessities.

**Figure 16:**
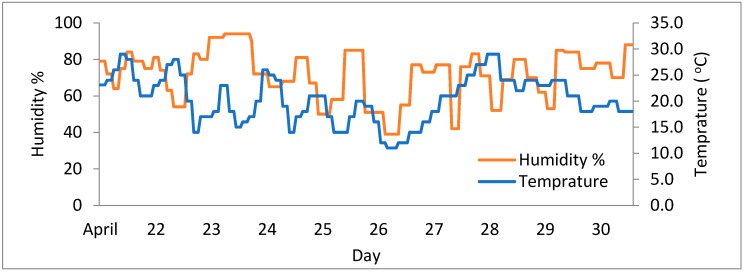
Relative humidity and temperature measurements.

**Figure 17:**
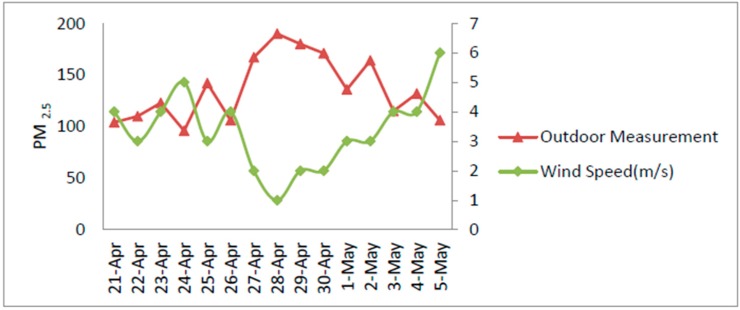
Effect of the wind speed on PM_2.5_ concentration (microgram per cubic meter).

PM_2.5_ concentrations are greatly linked to some of the atmospheric properties like wind speed, temperature and humidity. The effect of these parameters on PM_2.5_ concentrations was observed, Figure 16 display the relative humidity and temperature for the under-observation days. Figure 17 plots the associations among PM_2.5_ and the wind speed. It can be observed that from 28 April to 2 May the PM_2.5_ concentrations increased progressively with the wind speeds reaching approximately 3.5 m/s. Afterward the speeds dropped gradually and held in a lower range, was owing to the dispersal of the pollutants by strong winds [36]. It is possible to conclude that AQI depends on the wind speed variation to some extent as PM_2.5_ is a major participant of the AQI.

**Figure 18:**
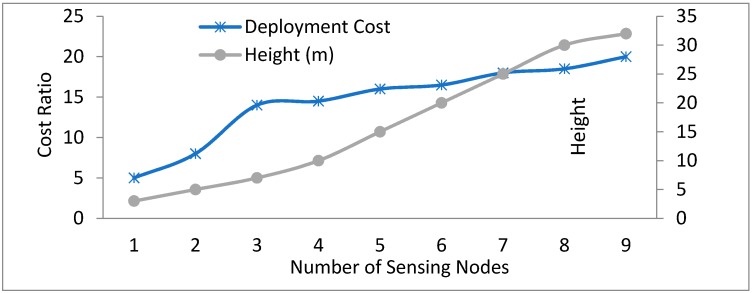
Relationship between number of the sensing nodes, node height and the deployment cost.

Figure 17 describes the impact of the number of sensing nodes and their positioned height on the overall deployment cost. The cost ratio ranges from 1 to 20. The addition of the sensing nodes to enhance the efficiency has a lower deployment cost as compared to the addition of sinks/ECD that are equipped with pollution sensors. Our monitoring system has designs corresponding to the mono-sink case. Graph shows that as the number of sensing nodes increases, they cause a rise in deployment costs. Similarly, the node’s deployment height also plays an important role in cost effectiveness. Trends shown in Figure 18 conclude the directly proportional relation between cost and the number of sensing node plus their placement height.

**Figure 19:**
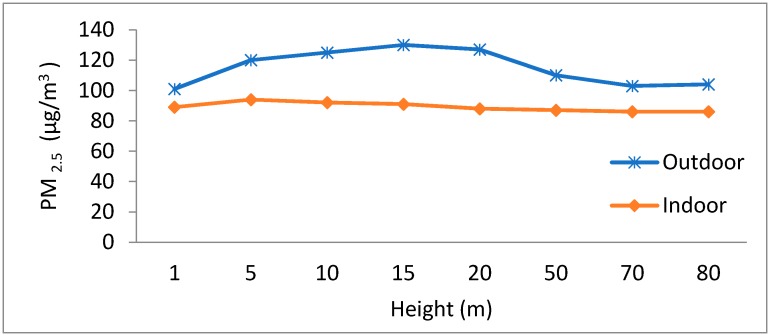
Effect of the node deployment height on PM_2.5_ concentration.

**Figure 20:**
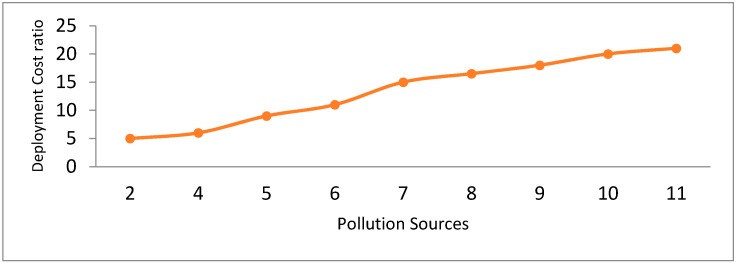
Impact of the pollution sources on the overall system deployment cost.

The impact of the sensing node height on the deployment cost and data accuracy was studied. For the data collection, experiments were conducted on the different floors of the international student dormitory building FUDAN University Handan Campus. The campus has 23 floors and a total height of approx. 90 m. Figure 19 reveals the effects of the optimal deployment height i.e., near major pollutants. This study is helpful in node positioning, and to estimate where it will provide the accurate value. From the trends, we observed that pollution concentration tends to rise near the ground 1–20 m and then start decreasing gradually. At high altitudes, pollution sources are more dispersed and have low concentration as compared to the ground level, especially in case of particle matters [24]. Figure 20 shows the overall deployment cost depending on the measurement height, it was observed that the deployment cost is nominal when the nodes height is near the effective release height of pollution sources, which was found to be around 9 m. This is explained by the fact that the pollution concentration becomes high when being near to the pollutant active release height, along with this the pollutants have a greater tendency to drop than to rise because of gravitational effects [23,24]. 

The incorporation of the more pollutants enhances the deployment cost significantly. As demonstrated in Figure 20, more pollutants results in a larger number of related sensors. Further sensors integration ultimately increases the deployment expenses. The rise in the cost ratio from 5 pollutants to 6 pollutants is smaller than the growth from 4 sources to 5. This is for the reason that increasing pollution sources results in many intersections between pollution zones [24], which directly affect the system costs.

Below is the summary of the features possessed by the proposed monitoring system and its comparison with existing systems (Table 3).

## 6. Conclusions

In this paper, we developed an edge-computing based IoT architecture for the air pollution monitoring system. The project integrates open access, low cost technologies. The system’s prototyping was done with a small size, cheap and easy to develop & deploy Arduino platform. Prototype is able to monitor multiples gases and particular matter along with humidity and temperature. Algorithms were employed to avoid temporary sensor errors and to manage the cross sensitivity problems. Automatic calibration was applied to ensure the accuracy of the sensors reporting. Another algorithm was developed to minimize the redundant network traffic and to reduce the power consumption. The designed model is deliberated in such a way to reduce the computational burden over sensing nodes that is battery operated and balanced the total work load with edge computing device. The proposed system has no limits on the installation location. Watson studio was employed to explore air quality data and to create the data visualizations for the end user. The study concluded that it is best to position the sensing node at the optimal height near to the pollutants. The system model is intended for static sensor networks, supported by the fact that the pollution sensors work well in static mode. A sufficiently number of experiments have been conducted in different locations to endorse the reliability of the air quality monitoring system. Various remarkable facts have been discovered when associating the air quality tendency and other similar statistics.

From the large volume of data, useful information was generated and published to the users at the local level to create awareness. This is an excellent solution for household, offices and crowded environment monitoring, although for industrial monitoring, current versions need to be upgraded for higher accuracy. Nodes are scalable and allow easy up gradation according to demand. 

## Figures and Tables

**Table 1:** Pollution monitoring systems and related requirements.

System Type	Deployment & Maintains	Cost	Accuracy	Power Consumption	Response Time
Indoor	Easy	Little	Average	Low	Average
Outdoor	Average	Average	High	Little	Average
Industrial	Average	Average	Very High	Average	Fast

**Table 2:** Summary of the related work.

System	Carrier	Communication Protocols	Sensing Node	Application Environment	Number of Sensing Nodes	Cost Estimation ($)
[17] 2017	NM	XBee Module, WIFI	Waspmote Board	Outdoor	Multiple	1250
[26] 2017	Lighting Pole	Ethernet, WIFI	Arduino	Outdoor	1	500
[27] 2017	NA	WIFI	Arduino	Indoor	1	600
[8] 2017	NM	NM	Raspberry Pi	Outdoor	1	1000
[20] 2017	Quad Copter	NM	NM	Outdoor	1	1500
[28] 2017	NM	WIFI	Raspberry Pi	Outdoor	1	1000
[29] 2017	NA	2.4 GHz ISM Band	STC12C5A60S2	Indoor	1	1500
[30] 2017	Roof Top	LTE	NM	Outdoor	Multiple	X
[16] 2017	Public	WIFI	ARM Mbed	Outdoor	Multiple	1000
[31] 2017	NA	Bluetooth, Ethernet	Arduino, Raspberry Pi	Indoor	1	1300
[6] 2017	Lighting Pole	Wi-Fi	Arduino	Indoor	1	500
[32] 2017	NM	Wi-Fi	Arduino, Lab View	Indoor	1	1200
[7] 2016	Mobile Sensors	Zigbee Module	Arduino, Raspberry Pi	Outdoor	1	1400
[9] 2016	Bus Top	NA	Mosaic, GPS	Outdoor	8	800
[14] 2016	Lighting Pole	Public Hotspot	Linux Embedded System	Outdoor	Multiple	1200
[33] 2016	Bus Top (Mobile Sensors)	NM	NM	Outdoor	1	500
[34] 2016	Public	GPRS	NM	Outdoor	Multiple	1000
[35] 2016	NA	Wi-Fi	Arduino	Indoor	1	1200
[10] 2016	NA	Wi-Fi, Bluetooth, RF	TIMSP430	Indoor	1	1200
[22] 2016	NM	MQTT	AVR Atmega128	Outdoor	1	700
[36] 2016	Lighting Pole	IEEE 802.15.4k	STM32F103RC Microcontroller Unit	Outdoor	Multiple	1000+
[24] 2017	Simulations	Simulations	IBM ILOG Data Set	Outdoor	Multiple	NA
[37] 2017	Simulations	Simulations	NA	Outdoor	Multiple	NA
[38] 2017	Simulations	Simulations	Time-Varying Data Sets	Outdoor	Multiple	NA

NA = Not Applicable. NM = Not Mentioned

**Table 3:** Comparison of the proposed and the existing pollution monitoring systems.

Features	Proposed Monitoring System	Models Presented in the Literature	Official Monitoring Systems
Cost	Low	High	Very High
Accuracy	Good-Average	Average-Low	Very Good
Power Consumption	Low	High	Very High
System Deployment	Easy	Complex	Highly Complicated
Maintains	Easy	Moderate	Difficult
Scalability & Up gradation	Yes	Mostly Not	Yes
Accessible to Common User	Yes	Yes	No
